# Toxicity of the Essential Oil of *Illicium difengpi* Stem Bark and Its Constituent Compounds Towards Two Grain Storage Insects

**DOI:** 10.1673/031.011.15201

**Published:** 2011-11-09

**Authors:** Sha Sha Chu, Cheng Fang Wang, Shu Shan Du, Shao Liang Liu, Zhi Long Liu

**Affiliations:** ^1^ Department of Entomology, China Agricultural University, 2 Yuanmingyuan West Road, Haidian District, Beijing 100094, China; ^2^ State Key Laboratory of Earth Surface Processes and Resource Ecology, Beijing Normal University, Beijing 100875, China; ^3^ Department of Biology, Faculty of Preclinical Medicine, GuangXi Traditional Chinese Medical University, Nanning 530001, China

**Keywords:** contact toxicity, fumigant, *Sitophilus zeamais*, *Tribolium castaneum*

## Abstract

During our screening program for new agrochemicals from Chinese medicinal herbs, the essential oil of *Illicium difengpi* stem bark was found to possess strong insecticidal activities against the maize weevil, *Sitophilus zeamais* (Motschulsky) (Coleoptera: Curculionidae) and red flour beetle, *Tribolium castaneum* Herbst (Coleoptera: Tenebrionidae). A total of 37 components of the essential oil of *I. difengpi* were identified. The main components of the essential oil were safrole (23.61%), linalool (12.93%), and germacrene D (5.35%). Bioactivities-directed chromatographic separation on repeated silica gel columns led to the isolation of two compounds: safrole and linalool. Safrole showed pronounced contact toxicity against both insect species and (LD_50_ = 8.54 for *S. zeamais*; 4.67 µg/adult for *T. castaneum*) and was more toxic than linalool (LD_50_ = 24.88 for *S. zeamais*; 8.12 µg/adult for *T. castaneum*). The essential oil acting against the two species of insects showed LD_50_ values of 13.83 and 6.33 µg/adult, respectively. Linalool also possessed strong fumigant toxicity against both insect species (LC_50_ = 10.02 for *S. zeamais*; 9.34 mg/L for *T. castaneum*) and was more toxic than safrole (LD_50_ = 32.96 and 38.25 mg/L), while the crude essential oil acting against the two species of insects showed LC_50_ values of 14.62 and 16.22 mg/L, respectively. These results suggest that the essential oil of *I. difengpi* stem bark and the two compounds may be used in grain storage to combat insect pests.

## Introduction

*Sitophilus* and *Tribolium* species are the major pests of stored grains and grain products in the tropics and subtropics. Control of stored product insects relies heavily on the use of synthetic insecticides and fumigants, which has led to problems such as disturbances of the environment, increasing costs of application, pest resurgence, pest resistance to pesticides, and lethal effects on non-target organisms in addition to direct toxicity to users (Isman 2004). Fumigation plays a very important role in insect pest elimination in stored products (Zettler and Arthur 2000). Plant essential oils and their components have been shown to possess potential to be developed as new fumigants and they may have the advantage over conventional fumigants in terms of low mammalian toxicity, rapid degradation, and local availability (Isman 2004, 2006). Essential oils derived from more than 75 plant species have been evaluated for fumigant toxicity against stored product insects so far (see review by Rajendran and Srianjini 2008).

Botanical pesticides have the advantage of providing novel modes of action against insects that can reduce the risk of cross-resistance as well as offering new leads for design of target-specific molecules (Isman 2006, 2008). During the screening program for new agrochemicals from Chinese medicinal herbs, the essential oil of *Illicium difengpi* B. N. Chang (Austrobaileyales: Schisandraceae) stem bark was found to possess strong insecticidal toxicity against the maize weevils, *Sitophilus zeamais* (Motschulsky) (Coleoptera: Curculionidae) and red flour beetles, *Tribolium castaneum* Herbst (Coleoptera: Tenebrionidae).

*Illicium difengpi*, indigenous to China, is a toxic shrub that grows in the mountainous areas of Guangxi Zhuang Nationally Autonomous Region. The stem bark is listed in Chinese Pharmacopoeia and has been applied as a traditional Chinese medicine to treat rheumatic arthritis (Editorial Committee of Chinese Pharmacopoeia 2005). To date, around 30 compounds including triterpene acids, phenylpropanoids, neolignans, and their glycosides were isolated from the stem bark of this plant (Kouno et al. 1992, 1993; Huang et al. 1996, 1997a, 1997b; Fang et al. 2010). The chemical composition of *I. difengpi* essential oil was also studied previously (Jui et al. 1981; Rui and Ji 1992; Wang et al. 1994; Liu et al. 1996). However, no reports on insecticidal activity of *I. difengpi* essential oil against stored product insects are available.

## Materials and Methods

### Chinese medicinal herb and extractions

Five kilograms of stem bark of *I. difengpi* was collected in October 2008 from Yulin City, Guangxi Autonomous Region (Guangxi 537000, China). A voucher specimen (CAU-Zhongyao-Difengpi-SB001) has been deposited in the Department of Entomology, China Agricultural University, Beijing 100094, China. Stem bark samples were air-dried and ground to powder using a grinding mill (Retsch Muhle, www.retsch.com). Each 600 g portion of powder was mixed into 1800 ml of distilled water and soaked for three hours. The mixture was then boiled in a round-bottom flask, and steam distilled for six hours. Volatile essential oil from distillation was collected in a flask. Separation of the essential oil from the aqueous layer was done in a separatory funnel, using the non-polar solvent *n*-hexane. The solvent was evaporated at 40° C using a BUCHI Rotavapor R-124 vacuum rotary evaporator (BUCHI, www.buchi.com). The sample was dried over anhydrous sodium sulfate and kept in a refrigerator at 4° C for subsequent experiments.

### Insects

The maize weevils (*S. zeamais*) and red flour beetles (*T. castaneum*) were obtained from laboratory cultures maintained in the dark in incubators at 29–30° C and 70–80% relative humidity. The red flour beetles were reared on wheat flour mixed with yeast (10:1 w/w) while maize weevils were reared on whole wheat at 12–13% moisture content. Unsexed adult weevils/beetles used in all the experiments were about two weeks old.

### Fumigant toxicity

A 2.0 cm diameter Whatman filter paper (Whatman, www.whatman.com) was placed on the underside of the screw cap of a glass vial measuring 2.5 cm diameter, 5.5 cm height, and 24 ml volume. Range-finding studies were run to determine the appropriate testing concentrations. Ten microliters of essential oil/compounds (six concentrations) were added to the filter paper. The solvent was allowed to evaporate for 15 seconds before the cap was placed tightly on the glass vial, containing 10 unsexed insects, to form a sealed chamber. Fluon was used inside the glass vial to prevent insects from the treated filter paper. *n*-Hexane was used as a control. Six replicates were used in all treatments and control and they were incubated at 29–30° C and 70–80% relative humidity. for 24 hours. The insects were then transferred to clean vials with some culture media and kept in an incubator. Mortality of insects was observed daily until end-point mortality was reached one week after treatment. Results from all replicates were subjected to probit analysis using the PriProbit Program V1.6.3 to determine LC_50_ values (Sakuma 1998).

### Contact toxicity using topical application

The contact toxicity of essential oil against *S. zeamais* and *T. castaneum* adults was measured as described by Liu and Ho (1999). Range-finding studies were run to determine the appropriate testing concentrations. A serial dilution of the essential oil/compounds (six concentrations) was prepared in *n*-hexane. Aliquots of 0.5 µl of the dilutions were applied topically to the dorsal thorax of the insects. Controls were determined using *n*-hexane. Six replicates were used in all treatments and controls. Both treated and control insects were then transferred to glass vials (10 insects/vial) with culture media and kept in incubators. Mortality of insects was observed daily until end-point mortality was reached one week after treatment. The LD_50_ values were calculated by using Probit analysis (Sakuma 1998). Positive control, pyrethrum extract (25% pyrethrine I and pyrethrine II), was purchased from Fluka Chemie (www.sigmaaldrich.com).

### Gas chromatography and mass spectrometry

Components of the essential oil were separated and identified by gas chromatography—mass spectrometry (GC—MS) on an Agilent 6890N gas Chromatograph (Agilent Technologies, www.agilent.com) hooked to Agilent 5973N mass selective detector. They were equipped with a flame ionization detector (280° C) and fitted with a HP-5MS column (30m × 0.25mm, df = 0.25 µm). The GC settings were as follows: initial oven temperature was held at 60° C for one minute and ramped at 10° C minute^-1^ to 180° C for one minute, and then ramped at 20° C minute^-1^ to 280° C for 15 minutes. The injector temperature was maintained at 270° C. The samples (1 µl) were injected neat, with a split ratio of 1:10. The carrier gas was helium at flow rate of 1.0 ml minute^-1^. Spectra were scanned from 20 to 550 m/z at two scans s^-1^ in EI mode at 70 eV. The constituents were identified by comparison of their retention indices with those of the literature (Rui and Ji 1992; Wang et al. 1994; Liu et al. 1996) or with those of authentic compounds available in our laboratories. The retention indices were determined in relation to a homologous series of *n*-alkanes (C_8_–C_24_) under the same operating conditions. Further identification was made by comparison of mass spectra with those stored in NIST 05 and Wiley 275 libraries, or with mass spectra from literature (Adams 2007). Component relative percentages were calculated based on GC peak areas for each component; the response factor was fixed to 1.

### Bioassay-directed fractionation

The crude essential oil (25 ml) was chromatographed on a Merck 9385 1,000g silica gel (Merck, www.merck.com) column (85 mm i.d., 850 mm length) by gradient elution with a mixture of solvents (*n*-hexane, *n*-hexane-ethyl acetate, from 100:1, 100:2, 100:5, …, 100:50). Fractions of 500 ml were collected and concentrated at 40° C, and similar fractions according to thin layer chromatography profiles were combined to yield 27 fractions. Fraction 5 to 11, which possessed contact/fumigant toxicity with similar thin layer profiles, were pooled and further purified by preparative silica gel column chromatography (*n*-hexane: ethyl acetate = 20:1) to obtain two pure compounds for determining structure as linalool (1.1 g), and safrole (1.6 g). 1H nuclear magnetic resonance (NMR) spectra were recorded on Bruker ACF300 (300MHz (1H))and AMX500 (500MHz (1H)) instruments (Bruker Corporation, www.bruker.com) using deuterochloroform (CDC13) as the solvent and tetramethylsilane as the internal standard. Electron impact mass spectra were determined on a Micromass (www.waters.com) VG7035 mass spectrometer at 70 eV.

**Figure 1.**
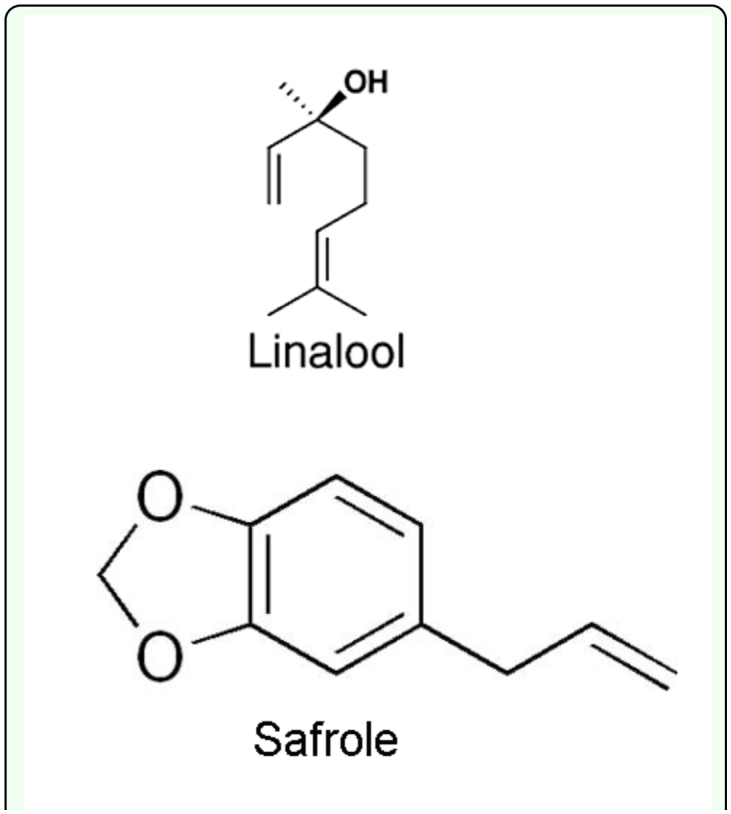
Structures of linalool and safrole. High quality figures are available online.

**Table 1.**
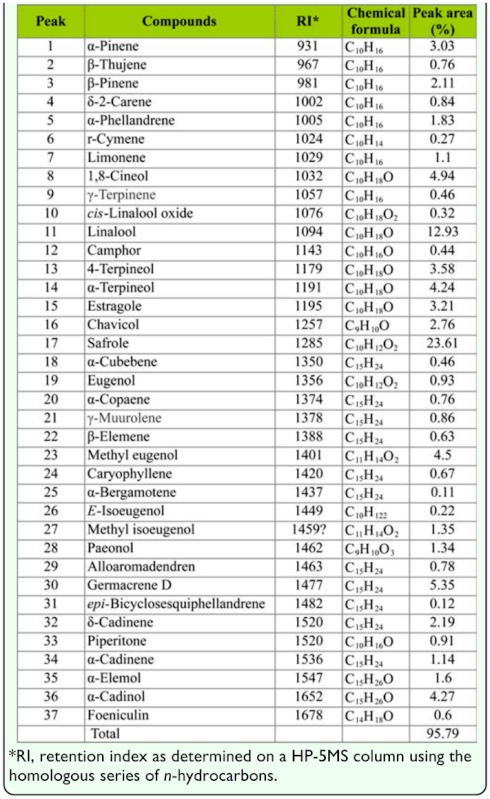
Chemical constituents of the essential oil derived from *Illicium difengpi* stem bark.

## Results

The six-hour steam distillation of *I. difengpi* stem bark afforded essential oil (yellow) with a yield of 0.13% (v/w); the density of the concentrated essential oil was determined to be 0.86 g/ml. The GC—MS analysis of the essential oil led to the identification and quantification of a total of 37 major components accounting for 95.79% of the total components present (Table 1). The main components of the essential oil were safrole (23.61%), linalool (12.93), and germacrene D (5.35%) (Figure 1).

Based on bioassay-guided fractionation, two compounds were separated and purified by column chromatography and preparative thin layer chromatography. The identifications were supported by the following data: Linalool (**1**), colorless oil, C_10_H_18_O, ^1^HNMR (300Hz, CDCl_3_) δ: 1.27 (3H, s, 10-CH_3_), 1.56 (2H, m, 4-CH_2_), 1.60 (3H, s, 8-CH_3_*), 1.68 (3H, s, 9-CH_3_*), 1.96 (2H, q, *J* = 0.02 Hz, 5-CH_2_), 2.02 (1H, 3-OH), 5.05 (1H, d, *J* = 0.032 Hz, 6-H), 5.18 (2H, 1-CH_2_), 5.90 (1H, dd, *J* = 0.035, 0.016 Hz, 2-H). * = assignments may be interchanged. ^13^CNMR (75Hz, CDCl_3_) δ: 145.2 (C-2), 131.7 (C-7), 124.5 (C-6), 111.7 (C-1), 73.4 (C-3), 42.2 (C-4), 27.8 (C-10), 25.7 (C-8*), 22.9 (C-5), 17.7 (C-9*). * = assignments may be interchanged. MS *m/z* (%): 154 (M^+^, 5), 136 (15), 121 (25), 109 (11), 93 (80), 83 (18), 80 (30), 71 (100), 69 (50), 55 (45), 43 (39), 41(62), 27 (15). The data matched with the previous reports from Bohlmann et al. (1975) and Phutdhawong et al. (2007).

**Table 2.**
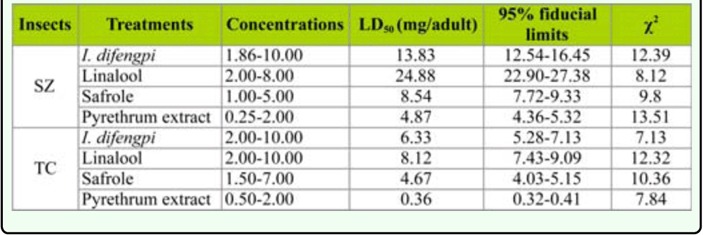
Contact toxicity of the essential oil of *Illicium difengpi* against *Sitophilus zeamais* (SZ) and *Tribolium castaneum* (TC) adults.

Safrole (**2**), slightly yellow oil, C_10_H_12_O_2_, ^1^H-NMR (300 MHz, CDCl_3_) δ: 6.78 (1H, d, *J* = 7.9 Hz, 5′-H), 6.72 (1H, d, *J* = 1.4 Hz, 2′-H), 6.67 (1H, dd, *J* = 7.9, 1.7 Hz, 6′-H), 5.96 (1H, m, 2-H), 5.95 (2H, s, O-CH_2_-O), 5.10 (2H, m, 1-CH_2_), 3.28 (2H, d, *J* = 6.7 Hz, 3-CH_2_). ^13^C-NMR (75Hz, CDCl_3_) δ: 147.7 (C-3′), 145.9 (C-4′), 137.7 (C-2), 134.3 (C-1′), 121.7 (C-6′), 115.6 (C-1), 109.5 (C-2′), 108.1 (C-5′), 101.2 (O-CH_2_-O), 39.9 (C-3). MS *m/z* (%): 163 (M^+^, 11), 162 (100), 161 (26), 135 (31), 132 (13), 131 (34), 105 (10), 104 (39), 103 (27), 78 (16), 77 (27), 51 (19). The data matched with previous reports from Sy et al. (1997) and Mohottalage et al. (2007).

Safrole showed stronger contact toxicity against *S. zeamais* (LD_50_ = 8.54 µg/adult) and *T. castaneum* (LD_50_ = 4.67 µg/adult) than linalool (LD_50_ = 24.88 and 5.12 µg/adult, respectively) while the essential oil of *I. difengpi* possessed contact toxicity against *S. zeamais* and *T. castaneum* with LD_50_ value of 13.83 µg/adult and 6.33 µg/adult, respectively (Table 2). However, linalool demonstrated stronger fumigant toxicity to *S. zeamais* (LC_50_ = 10.02 mg/L air) and *T. castaneum* adults (LC_50_ = 9.34 mg/L air) than safrole (LC_50_ = 32.96 and 38.25 mg/L air, respectively). The essential oil possessed strong fumigant activity against *S. zeamais* and *T. castaneum* adults with LC_50_ value of 14.62 mg/L air and 16.22 mg/L air, respectively (Table 3).

**Table 3.**
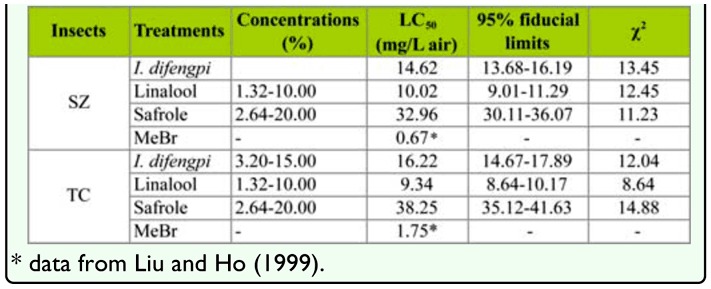
Fumigant toxicity of the essential oil of *Illicium difengpi* against *Sitophilus zeamais* (SZ) and *Tribolium castaneum* (TC) adults.

## Discussion

GC-MS results showed that the main components of *I. difengpi* stem bark essential oil were safrole, linalool, and germacrene D. In the previous reports, safrole and linalool were also demonstrated to be the two major constituents of the essential oil although the content of them varied in some degrees. For example, Rui et al. (1984) found that the essential oil of *I. difengpi* stem bark contained safrole (30.99%), linalool (13.16%), 1,8-cineol (8.19%), β-pinene (8.03%), camphor (7.56%), and α-pinene (6.35%). Another report (Rui and Ji 1992) indicated that the major components of the essential oil of *I. difengpi* stem bark were safrole (21.74%), linalool (15.51%), 1,8-cineol (8.77%), benzene 1,2-dimethoxy-4-(2-propenyl) (6.30%), and α-terpineol. Moreover, Liu et al. (1996) reported that the essential oil of *I. difengpi* stem bark contained safrole (28.64%) and linalool (16.83%), followed by 1,8-cineol (4.74%) and camphor (4.52%). Moreover, there were some variations in the essential oils derived from different parts of *I. difengpi*. For example, the essential oil of *I. difengpi* fruits possessed limonene (9.54%), 1,8-cineol (9.00%), and α-calacorene (8.29%) (Wang et al. 1994). However, the essential oil of *I. difengpi* leaves contained safrole (43.31%), linalool (16.58%), 1,8-cineol (8.77%), β-pinene (7.40%), and α-pinene (5.73%) (Rui et al. 1984). The above results suggest further studies on plant cultivation and essential oil standardization are needed.

The essential oil of *I. difengpi* stem bark showed contact toxicity against *S. zeamais* and *T. castaneum* (Table 2). However, the essential oil demonstrated 3 and 18 times less acute toxicity against the two species of insects when compared with the positive control (pyrethrum extract, 25% pyrethrine I and Pyrethrine II) because the pyrethrum extract has acute toxicity to *S. zeamais* and *T. castaneum* with LD_50_ value of 4.87 µg/adult and 0.36 µg/adult, respectively (Table 2). The isolated compounds also possessed weaker contact toxicity against the species of insects when compared with the positive control (Table 2).

The essential oil of *I. difengpi* stem bark and the two compounds also possessed strong fumigant activity against *S. zeamais* and *T. castaneum* adults (Table 3). The currently used grain fumigant, methyl bromide (MeBr), was reported to have fumigant activity against *S. zeamais* and *T. castaneum* adults with LC_50_ values of 0.67 mg/L and 1.75 µg/L air, respectively (Liu and Ho 1999). Compared with the commercial fumigant MeBr, the essential oil of *I. difengpi* stem bark was 22 and 9 times less toxic to *S. zeamais* and T. *castaneum*, respectively. However, compared with the other essential oils in other studies, the essential oil exhibited the same level of fumigant toxicity against maize weevils, i.e., essential oils of *Murraya exotica* (LC_50_ = 8.29 mg/L) (Li et al. 2010), *Artemisa lavandulaefolia* LC_50_ = 11.2 mg/L), *A. sieversiana* (LC_50_ = 15.0 mg/L) (Liu et al. 2010), *A. vestita* (LC_50_ =13.42 mg/L) (Chu et al. 2010a) and *Illicium simonsii* (LC_50_ = 14.95 mg/L) (Chu et al. 2010b). Compared with MeBr, linalool was 15 and 5 times less toxic to the two species of insects, respectively. However, considering the currently used fumigants are synthetic insecticides, fumigant activity of the essential oil and the two isolated compounds is quite promising.

Linalool was demonstrated to possess insecticidal activity against the second instar larvae of gypsy moth (*Lymantria dispar*) (Kostic et al. 2008) adults of the three tephritid fruit fly species (*Ceratitis capitata*, *Bactrocera dorsalis*, and *Bactrocera cucurbitae)* (Chang et al. 2009). It was also found to have fumigant toxicity against the triatomine bug (*Rhodnius prolixus*) (Sfara et al. 2009) and the housefly with a 24 hour LC_50_ value of 13.6 mg/L air (Palacios et al. 2009). Moreover, linalool possessed both contact and fumigant toxicity against human head louse (*Pediculus humanus capitis*) (Yang et al. 2009), and showed a high acaricidal activity by vapor action against mobile stages of *Tyrophagus putrescentiae* (Sanchez-Ramos and Castanera 2001). Linalool was found to be a competitive inhibitor of acetylcholinesterase (AchE) (Ryan and Byrne 1988).

As for the other isolated compound, safrole was shown to have both contact and fumigant toxicity against several insect species and mites in previous studies. For example, Ngoh et al. (1998) measured contact and fumigant toxicity as well as repellency activity against the American cockroache, *Periplaneta Americana*, while Huang et al. (1999) determined contact and fumigant toxicity as well as feeding deterrent activity against two storage insects (*T. castaneum* and *S. zeamais*). Safrole was found to possess fumigant toxicity against the adult housefly, *Musca domestica,* with a LC_50_ value of 4.8 mg/L (Mohottalage et al. 2007). It also was showed to be toxic to house dust mites, *Dermatophagoides farinae*, and *D. pteronyssinus* (Cho et al. 2004). Moreover, safrole also possessed larvicidal activity against two mosquitoes, *Aedes aegypti* and *Ae. albopictus* (Jantan et al. 2005; Cheng et al. 2009), as well as the tobacco armyworm, *Spodoptera litura* (Bhardwaja et al. 2010). However, safrole was demonstrated to be anticipated as a human carcinogen based on sufficient evidence of carcinogenicity from studies in experimental animals (United States Department of Health and Human Services 2011). This may limit the practical use of safrole.

These findings, considered together, suggest that the essential oil of *I. difengpi* stem bark and the two compounds show potential to be developed as natural insecticides/fumigants for control of stored product insects. However, for the practical application of the essential oil and the two compounds as novel insecticides/fumigants, further studies on the safety of the essential oil and the two compounds toward humans and on the development of formulations are necessary to improve the efficacy and stability, and to reduce cost.

## Abbreviations:

GC: gas chromatography;
MS: mass spectrometry;
NMR: nuclear magnetic resonance
